# Phage Therapy Experience at the Eliava Phage Therapy Center: Three Cases of Bacterial Persistence

**DOI:** 10.3390/v13101901

**Published:** 2021-09-23

**Authors:** Elisabed Zaldastanishvili, Lika Leshkasheli, Mariam Dadiani, Lia Nadareishvili, Lia Askilashvili, Nino Kvatadze, Marina Goderdzishvili, Mzia Kutateladze, Nana Balarjishvili

**Affiliations:** 1 Laboratory of Molecular Biology, Eliava Institute of Bacteriophages, Microbiology and Virology, 0160 Tbilisi, Georgia; l.leshkasheli@pha.ge (L.L.); laskilashvili@pha.ge (L.A.); n.balarjishvili@pha.ge (N.B.); 2 Eliava Phage Therapy Center (EPTC), 0160 Tbilisi, Georgia; mari.dadiani@gmail.com (M.D.); nadalia30.60@gmail.com (L.N.); 3 Laboratory of General Microbiology, Eliava Institute of Bacteriophages, Microbiology and Virology, 0160 Tbilisi, Georgia; nkvatadze27@gmail.com (N.K.); mgoderdzishvili@pha.ge (M.G.); 4 Eliava Institute of Bacteriophages, Microbiology and Virology, 0160 Tbilisi, Georgia; kutateladze@pha.ge

**Keywords:** phage therapy, *Pseudomonas aeruginosa*, *Klebsiella pneumoniae*

## Abstract

In this retrospective descriptive study we focus on cases of three patients who underwent phage therapy procedures at Eliava Phage Therapy Center (EPTC) in Tbilisi, Georgia. Patients with chronic infectious diseases related to *Pseudomonas aeruginosa* (two patients, lower respiratory tract infection (LRTI)) and *Klebsiella pneumoniae* (one patient, urinary tract infection (UTI)) are among those very few EPTC patients whose pathogens persisted through phage therapy. By looking at bacterial strains and personalized phages used against them we tried to point towards possible adaptation strategies that are employed by these pathogens. Genome restriction-based Pulsed Field Gel Electrophoresis (PFGE) profiling of strains isolated before and after phage therapy hints towards two strategies of adaptation. In one patient case (*Pseudomonas aeruginosa* related lung infection) bacterial strains before and after phage therapy were indistinguishable according to their PFGE profiles, but differed in their phage susceptibility properties. On the other hand, in two other patient cases (*Pseudomonas aeruginosa* related LRTI and *Klebsiella pneumoniae* related UTI) bacterial adaptation strategy seemed to have resulted in diversification of infecting strains of the same species. With this work we want to attract more attention to phage resistance in general as well as to its role in phage therapy.

## 1. Introduction

As antibiotic-resistance remains an ever-growing concern, phage therapy has already received an unprecedented amount of attention [1]. While it has long been an acknowledged therapy option in many Eastern European and post-Soviet countries, the field is still short of successful clinical trials [2,3]. High costs related to executing clinical trials have hindered fast accumulation of much-needed data in the phage therapy field. Although no adverse effects have been associated with phage therapy so far, its effectiveness still needs to be demonstrated in clinical trials [4,5]. The two most recent reports from randomized, placebo-controlled, double-blind clinical trials showed no significant advantage of readily available phage preparations over placebo [5,6]. Therefore, reporting individual clinical cases, especially of patients who have received individualized phage preparations, has remained the method of choice among many researchers focusing on phage therapy [7,8,9,10,11,12,13].

Here we discuss three patient cases from the Eliava Phage Therapy Center (EPTC). These cases were selected upon examination of EPTC clinical data from the years 2018 and 2019. As most EPTC patients receive their treatment plans remotely, taking their anamneses is somewhat problematic. For this reason, here we concentrate on patients who have been physically attended to by EPTC clinicians in Tbilisi. However, it must be noted that even in such cases, patients’ medical histories still lack substantial information about treatments that they have received prior to and after their stays at EPTC.

The three cases discussed here were selected from among those cases in which bacterial clearance was followed up. The reason we concentrated on these cases is the observed persistence of bacteria. Although two out of the three patients discussed in this work report substantial progress in the development of their diseases, bacteria, which were targeted by antibiotics as well as by phages, remained present throughout the years. It must be noted here that the two patients whose conditions were improved despite the near-constant presence of bacterial pathogens are genetically predisposed to lower respiratory tract infections (LRTI). In the three cases, infectious diseases were associated with the presence of *Pseudomonas aeruginosa* (two cases, LRTI) or *Klebsiella pneumoniae* (one case, urinary tract infection (UTI)). Despite not being closely related, these two bacteria have some common traits. They both belong to the so-called ESKAPE group of pathogens, which are notorious for causing nosocomial infections and for their concerning antibiotic-resistance properties [14]. Eradicating these agents is becoming hard in an ever-growing number of clinical cases. Subsequently, their carriers often suffer from chronic diseases.

EPTC clinicians rely greatly on six readily available phage preparations which are manufactured by Eliava Biopreparations—a company affiliated with the G. Eliava Institute of Bacteriophages, Microbiology and Virology (IBMV). These preparations consist of phages active against *Staphylococci* (including *Staphylococcus aureus*), *Streptococci*, *Enterococci*, *Shigellae*, *Salmonellae*, *Protei*, *Escherichia coli* (including Enterohaemorrhagic *Escherichia coli*—EPEC) and *Pseudomonas aeruginosa*. Four preparations, which have been used in cases discussed in this work are listed in Table 1.

**Figure 1:**
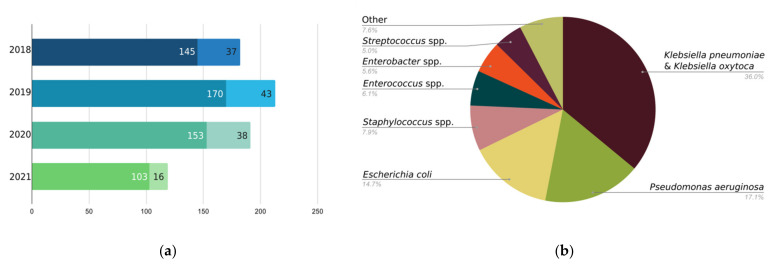
EPTC custom phage orders according to years (**a**) and bacterial species targeted by requested custom phages (**b**). Three full years and 6 months of the year 2021 are shown. Values in white (left part of the bar—darker shade of bar color) indicate numbers of primary custom phage orders, while values in black (right part of the bar—lighter shade of bar color) indicate numbers of custom phage re-orders from the same patients (**a**). Bacterial species distribution is shown cumulatively for 705 custom phage orders received since 2018 (**b**).

In cases when patients’ strains are not susceptible to the commercially available preparations, or if their infection is caused by an entirely different organism (on a species or genus level), an individualized phage preparation—custom phage—is offered. Such tailored bacteriophages are targeted at specific strains that have been isolated and identified in patients’ biological samples. This approach has proven successful for many patients suffering from different infectious diseases [8,11,13] and demand for custom phages is growing (Figure 1). While custom phage requests at EPTC differ in terms of targeted bacterial species from similar experiences elsewhere, increased interest in phage therapy seems to be a global, rather than a local trend [15,16].

It has long been known that bacteria possess several mechanisms of phage resistance [17]. Considering the matter of viral flexibility, compared to antibiotic resistance, phage resistance in bacteria is of much less concern. While still rare, phage resistance is encountered in clinic [18]. Therefore, its presence and abundance in nature highlight the need for better treatment opportunities in those rare cases, when patients are left with phage-resistant bacterial populations. In this paper, we try to bring such cases to the attention of wider scientific and medical communities. Investigating the aspects of phage–bacterial interactions that determine the treatment outcome could greatly improve our future approaches to phage therapy.

## 2. Materials and Methods

Isolation and identification of bacterial strains from clinical material was routinely performed at Eliava Analytical-Diagnostic Center. API^®^ systems (bioMérieux) were used for biochemical identification of bacterial species.

Bacterial strains were routinely grown in LB medium. Phage susceptibility was examined by spotting phage preparations on soft agar bacterial lawns as described (spot-test) [19]. Clear or semi-clear lysis in drop area was interpreted as phage sensitivity (S); opaque or turbid lysis in drop area was interpreted as intermediate susceptibility (I); no visible lysis in drop area was interpreted as phage resistance (R).

Bacterial cultures grown overnight were used for pulsed field gel electrophoresis (PFGE) plug preparation. Plug preparation, restriction digest as well as electrophoresis procedures were done according to the manufacturer’s instructions [20]. Plugs were casted in 1% PFGE grade SeaKem Gold agarose (Lonza) gel. Casted bacterial DNA was digested with *Xba*I (*Klebsiella pneumoniae* strains) and *Spe*I (*Pseudomonas aeruginosa* strains) restriction endonucleases (New England Biolabs). Electrophoresis was carried out at 14 °C in 0.5× TBE (130 mM Tris, 45 mM boric acid, 2.5 mM EDTA) for 18 h. Gel was visualized with ethidium bromide. Band analysis and tree generation was done with Freetree and TreeView software programs. Strains were clustered according to their band distributions based on un-weighted pair group method using average linkages (UPGMA). Scale bars in tree visualizations correspond to approximate genetic distances.

## 3. Results

### 3.1. Patient #1

Patient #1 (43 years old, male with cystic fibrosis) had been managing his lung infection with intravenous antibiotics since childhood. With the development of antibiotic resistance, disease management became difficult. According to the patient, the antibiotic plan, which was targeting *Pseudomonas aeruginosa*, had been failing for several years. Therefore, his main goal upon approaching EPTC was the replacement of antibiotics with an alternative treatment option. *Pseudomonas aeruginosa* had been confirmed as the main causative pathogen before visiting EPTC as well as in nine bacteriological analyses performed between January 2017 and September 2020. *Pseudomonas aeruginosa* detected in the first culture at Eliava Analytical-Diagnostic Center was susceptible to Pyo Bacteriophage and Intesti Bacteriophage, both of which contain phages active against *Pseudomonas aeruginosa* (Table 2). The patient started phage therapy on 7 January 2017. The initial treatment, which included daily administration of Pyo and Intesti Bacteriophages (each once a day, 8 mL taken orally and 2 mL via nebulizer) continued for 20 days. No complications were reported, and the patient was in a stable condition at the end of this 20-day course. After finishing the course, the patient was discharged on 27 January 2017 with a recommendation to continue year-round daily Pyo and Intesti bacteriophage inhalations via nebulizer complemented with 20-day treatment plan once every 3 months—when the same phages were administered orally. Sputum culture was regularly examined and at no instance was *Pseudomonas aeruginosa* absent. At different time-points, patient’s sputum culture inconsistently contained *Staphylococcus aureus*, *Staphylococcus epidermidis*, *Staphylococcus chromogenes*, *Streptococcus mitis* and/or *Streptococcus oralis* (Table 2).

As *Pseudomonas aeruginosa* strains of patient #1 slowly became resistant to Pyo and Intesti bacteriophages, the patient was offered an individualized phage preparation. The patient agreed, and in April 2019 he received custom phage—a phage preparation tailored for his specific strain of *Pseudomonas aeruginosa*. However, it must be noted, here, that later that year he ordered another custom phage as his sputum culture started to show resistance to the first custom phage soon after the start of its administration. Treatment with the second custom phage began in October 2019 and continued into the year 2020. Custom phages were administered daily and only via nebulizer. Two 20-day courses (with a two-week interval) were completed. Oral administration of Pyo and Intesti Bacteriophages was continued regularly even after resistance emerged in 2019. Interestingly, at the end of 2019, after four 20-day courses (with two-week breaks) were completed, this patient’s *Pseudomonas aeruginosa* culture once again started to respond to Pyo and Intesti bacteriophages.

In November 2019, the patient visited EPTC for the second time. According to him, since starting phage therapy in 2017, he resorted to taking antibiotics only once—in 2019, when his condition was worsened by a viral infection and when at the same time he had shortage of phages. Whether this incident could be linked to either the emergence of phage resistance or to its loss is hard to conclude.

Despite the fact that every examined sputum sample consistently contained *Pseudomonas aeruginosa*, it is important to mention that at the most recent examination, bacterial load was 10 to 100 times less compared to that of cultures collected previously (Table S1). This phenomenon was coincidentally observed only after the start of custom phage administration. As of 2020, the patient was able to completely replace antibiotics by phages.

**Figure 2:**
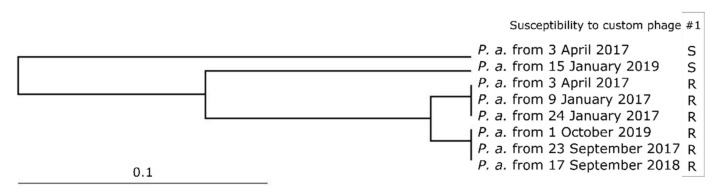
Clustering of *Pseudomonas aeruginosa* strains of patient #1. Susceptibility to the custom phage is shown across the isolation date of each strain. Cluster 1: *P. a.* from 3 April 2017b, *P. a.* from 9 January 2017, *P. a.* from 24 January 2017. Cluster 2: *P. a.* from 1 October 2019, *P. a.* from 23 September 2017, *P. a.* from 17 September 2018.

Eight strains of *Pseudomonas aeruginosa*, which had been collected before 2020, were compared in terms of their genetic relatedness (PFGE-based restriction profiles, Figure S1). While two small clusters (three strains each) of non-distinguishable strains were pronounced, it can be stated that the composition of this patient’s bacterial population varied over time (Figure 2). In this case, clustering seems to reflect strains’ phage susceptibility properties rather than their isolation dates (Table S2). All strains that showed resistance to the custom phage are clustered into two groups—both distant from the two sensitive strains. Interestingly, one of the sensitive strains was isolated together with a resistant strain (both from 03.04.2017). This fact allows us to presume that sensitive and resistant populations coexist, but sampling and isolation allows detection of the populations which were dominant at the time of sample collection. To the best of our knowledge this patient did not have any *Pseudomonas aeruginosa*-free episodes in the examined timeframe. Therefore, we believe it less likely that population diversity in this case stemmed from the acquisition of new pathogens after eradicating previous ones. Post-phage treatment strain’s (strain from 1 October 2019) close genetic relatedness to strains from two years ago favors the assumption that this strain too had been present before custom phage administration. While the strain that was used for custom phage preparation (strain from 15 January 2019) may have been successfully eradicated by the custom phage, as we have just found out, its distant resistant relatives were unfortunately not targeted.

### 3.2. Patient #2

Not unlike patient #1, patient #2 (64 years old, female with primary ciliary dyskinesia, bronchiectasis, refused antibiotic therapy options) had also been managing her infections with antibiotics for many years. At EPTC, her main motivation was antibiotic replacement with phages. Pyo and Intesti phages could not be considered an only option this time as from the very first culture, this patient’s *Pseudomonas aeruginosa* showed resistance to both of these products (Table 3). Therefore, custom phage was ordered the same summer and the patient started custom phage therapy in September 2018 (Table S4). Custom phage was administered orally—twice daily for 20 days. For the management of Staphylococcal co-infections, custom phage was complemented with Staphylococcal Bacteriophage, which was taken orally once every day for 20 days. As the patient could not tolerate the nebulizer, phages, including the custom phage, were administered only orally. A total of four courses were conducted.

Custom phage resistance was recorded for the first time in November 2018. Interestingly, later, *Pseudomonas aeruginosa* strain isolated from the 11 March 2019 sample no longer showed resistance to the first custom phage. Regardless, the patient ordered a second custom phage in April 2019 and started its application already in June 2019. She underwent three 20-day courses (flanked by two-week intervals) of oral administration of 10 mL of custom phage twice a day.

The following sputum culture from 31 July 2019 is interesting because of the presence of two phenotypically distinct strains of *Pseudomonas aeruginosa*. These two strains, when grown on Pseudomonas Isolation Agar (PIA), differed in their pigmentation: one variant (31 July 2019a) had greenish-blue colonies, usually associated with *Pseudomonas aeruginosa*, while the second variant (31 July 2019b) had pale pinkish colonies, presumably indicating lack of pyocyanin production abilities. As later in vitro examination showed, one of these strains was sensitive to two custom phages used before its culturing and one custom phage, which the patient ordered in September 2019. The other strain from the same sampling, as well as the new culture from 2 September 2019, showed resistance to all three custom phages.

Post-treatment examination of *Pseudomonas aeruginosa* strains that had been isolated before 2020 showed that, regardless of their phage-resistance properties or any phenotypic differences, none of the seven strains differed in terms of their genome restriction profiles on PFGE (Figure S2). Therefore, all strains were clustered in one group and no tree was generated.

As her strains were being examined, the patient continued her phage treatment plan and she ordered her fourth and fifth custom phages in February 2020 and in September 2020, respectively. She received another batch of her most recent custom phage preparation in April 2021 and continues to manage her infection with phages only. The most recent (end of July 2021) bacteriological analysis of her sputum sample (which was brought to our attention shortly before the submission of this manuscript) did not detect *Pseudomonas aeruginosa*.

### 3.3. Patient #3

Patient #3 (72 years old, female with chronic bacterial cystitis), who suffered from *Klebsiella pneumoniae*-related recurrent UTI, approached EPTC with the intention of eradicating her pathogens. She first addressed EPTC in January 2018 (Table S3). According to her, her UTI symptoms first appeared in May 2017. The patient sees the connection between the beginning of her symptoms and the bladder catheterization that she underwent during past surgery (total mastectomy of the left breast). Her symptoms included pelvic discomfort, frequent urination and pain and burning sensation during and after urination. She reported frequent exacerbations (about 8–10 times during the year before approaching EPTC) and was treated with antibiotics without any success. *Klebsiella pneumoniae* was confirmed as the main UTI causative agent bacteriologically in January as well as in March 2018 (Table 4). This is when she ordered her first autophage.

In June 2018, Patient #3 arrived in Tbilisi. At that time, *Klebsiella pneumoniae* was also confirmed present in her vaginal swab, which was taken as part of her routine examination. Cystitis and bacterial vaginitis were diagnosed. The patient immediately started her 20-day phage therapy course (2 weeks at EPTC and one week after leaving Tbilisi). Custom phage was administered twice daily orally (20 days), once a day via vaginal suppositories (for 10 days) together with once-a-day oral administration of Intesti phage (20 days). While Intesti phage does not contain any components targeting *Klebsiella pneumoniae*, it is active against *Enterococcus faecalis* and *Escherichia coli*, which were present in the patient’s urine and vaginal samples by the start of her two-week stay at EPTC (Table 4). Upon leaving Tbilisi, patient #3 was recommended to take a two-week break after completing the first phage therapy course, followed by a 15-days of oral administration of custom phage (twice a day), Intesti phage (once a day) and custom phage-containing vaginal suppositories (10 days). This 15-day course was to be repeated again after a two-week break. *Klebsiella pneumoniae* was no longer found 11 days from the start of the initial treatment. However, on 9 October 2018, which was supposed to be the last day of the recommended treatment plan, *Klebsiella pneumoniae* was again present in her urine as well as in her vaginal swabs. This was the case in January 2019 as well. In 2019 the patient ordered another custom phage which she received in April 2019, when she was visiting EPTC. She left Tbilisi with a recommendation to administer her custom phage twice a day for 20 consecutive days in parallel with once-a-day oral administration of Intesti phage for a month. This recommendation included 10-day administration of custom phage in form of vaginal suppositories followed by a 6-day course of vaginal suppositories containing metronidazole, miconazole, extract of Centella asiata, polymixin B and neomycin (Table S5). The recommendation given to patient #3 by her clinicians included a one-month break, after which she was to renew her 20-day course of once daily oral administration of Intesti and Ses bacteriophages (in parallel) together with a 10-day course of Intesti vaginal suppositories for the management of co-infections. Follow-up bacteriology in July 2019 confirmed the presence of *Klebsiella pneumoniae* in urine sample and in vaginal swabs. Eradication of *Klebsiella pneumoniae* was not achieved. Patient #3 did not find the treatment helpful, and she did not come back to EPTC either in 2019 or later in 2020 or 2021.

**Figure 3:**
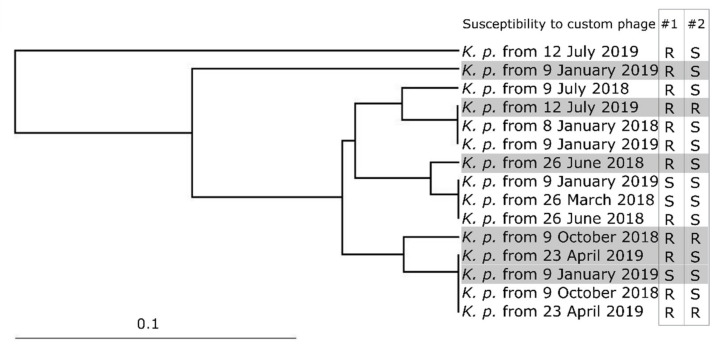
Clustering of *Klebsiella pneumoniae* strains of patient #3. Vaginal isolates are boxed in grey. Susceptibility to custom phages is shown across the isolation date of each strain. Cluster 1: *K. p.* (vaginal isolate) from 12 July 2019, *K. p.* from 8 January 2018, *K. p.* from 9 January 2019. Cluster 2: *K. p.* from 9 January 2019, *K. p.* from 26 March 2018, *K. p.* from 26 June 2018. Cluster 3: *K. p.* from 23 April 2019 (vaginal isolate), *K. p.* from 9 January 2019 (vaginal isolate), *K. p.* from 9 October 2018, *K. p.* from 23 April 2019.

PFGE examination (Figure S3) of this patient’s *Klebsiella pneumoniae* strains detected three clusters of very similar strains (each consisting of three or four members), three strains each closely related to one of these three clusters and two other distantly related strains (Figure 3). Interestingly, the clustering pattern reflects neither strain origin (urine or vaginal swab) nor strain isolation date. Members of the same cluster stem from different sampling time-points and do not share 100% similarity in their phage resistance properties.

Not unlike their UTI-notorious distant relatives, *Escherichia coli*, *Klebsiella pneumoniae* are also known to exploit their adhesion and invasion properties for long-term urinary tract persistence [21,22]. It is, therefore, logical to assume that in this patient’s case, too, new strain acquisition is unlikely and that already-present distinct strains have been replacing each other for the dominance of populations.

## 4. Discussion

The three cases discussed here (summarized in Table 5) can be split into two separate groups. Apart from the fact that an easy differentiation between the two bacterial species allows us to distinguish the pathogens and diseases, the patients’ main motivations also differ. Despite the fact that long-term total bacterial clearance was not achieved in any of the discussed cases, patients #1 and #2, both colonized with *Pseudomonas aeruginosa*, accomplished their goals of switching from antibiotics to phages with no adverse effects and with subjective alleviation of their symptoms. Therefore, these two cases can be grouped together. Though very similar in their management and outcomes, in terms of bacterial populations, these two cases differ fundamentally. Infections in the case of patient #1 were caused by strains which differ from each other. We presume, that this patient’s response to the treatment was diversification of its bacterial populations, including changing of the dominant culture.

Patient #2, on the other hand, had a strictly defined single population with stable resistance to Pyo and Intesti phages and with fast adaptation properties allowing the development of resistance to custom phages. Despite no significant changes in bacterial load (except for the very recently reported absence of *Pseudomonas aeruginosa*), this patient, too, achieved of the goal of antibiotic replacement. It is probable that both of these patients’ infections started with colonization by a single strain. In one case, the strain either gave rise to a diverse progeny, or new strains were acquired and incorporated in the existing population. In another case, the population remained uniform. Phage resistance acquisition was possible in both cases.

While on one hand we have patients, who are genetically predisposed to respiratory tract infections, on the other hand we have a patient with recurring urinary tract infection. Although there are reports of genetic background determining UTI susceptibility [23], chronic UTIs are still largely attributed to nonhereditary factors [24]. Therefore, we discuss this patient separately from the two mentioned above.

The first truly successful drug for treating UTI was Nitrofurantoin—first introduced in the early 1950s. Throughout the following decades, emerging antibiotic resistance led to its replacement first by beta lactams, then Trimethoprim/Sulfamethoxasole and finally by fluoroquinolones [25]. As this trend continues, alternatives are being actively sought. This includes alternatives for an ever-increasing incidence of UTI-causing multi-resistant *Klebsiella pneumoniae* strains. That is how and why patient #3 reached out for phage therapy. Despite the fact that the pathogen and the disease of patient #3 differ drastically from those of patient #1, if we regroup the three patients according to their bacterial population diversity, patients #1 and #3 end up together. In both of these cases it is very hard to determine a pattern in PFGE-based clustering of examined strains. In addition, as in the case of patient #1, in the case of patient #3, too, it seems that the pathogen’s main persistence strategy was population diversification. Apart from the potential acquisition of phage resistance and/or acquisition of a new strain, we cannot rule out the possibility that phage therapy enabled initially residing less susceptible strains to give rise to dominant bacterial populations.

We have encountered two more cases of *Klebsiella pneumoniae*-related persisting UTIs, where bacterial populations seemed to have changed (according to their PFGE profiles) not long after starting custom phage administration. While neither of these two patients received their treatments on site at EPTC, we lacked substantial part of their anamneses and decided not to discuss these cases in much detail. Despite not being able to follow these particular patient cases, we think that the pattern is similar to what we saw in patient #3. Therefore, we think that population diversification-driven persistence is not rare in *Klebsiella pneumoniae*-related UTI. Still, it is hard to draw any generic conclusions based on a couple of UTI cases. To find out whether switching predominant populations is common in UTIs in general, we plan to analyze UTI cases related to the most common cause of such infections—*Escherichia coli*. Hopefully, with the recession of COVID-19-related global restrictions, more patients will be able to travel to EPTC and many will be willing to contribute to this research.

The great majority of EPTC patients are from countries other than Georgia. Distance plays an important role in their decision whether to receive the treatment on-site in Tbilisi or to order phages remotely and to administer them according to EPTC doctors’ recommendations received through their telemedicine service. On one hand, telemedicine makes phage therapy accessible to a wider community; on the other hand, it has its own shortcomings. Among such limitations are difficulties in following up every single case and every single therapy course. Even those patients who physically visited EPTC and underwent full therapy courses on-site were discharged with recommendations, the fulfillment of which is hard to track. Of course, this is the reason behind the main shortcoming of this work—the absence of detailed information about antimicrobial treatments that these patients had received before and, in some cases, after phage therapy courses. These circumstances do not allow us to regard this paper as a classical case report. However, we believe that by such a preliminary screening of persisting bacterial strains, we will lay a foundation for more in-depth studies of phage resistance in clinic. To verify our assumption that bacterial populations adapt different strategies for resisting phages, we plan to further study bacterial strains and custom phages targeting them. We hope that by closely examining their genomes, we will be able to pinpoint the exact mechanisms enabling these strains to withstand phages in cases when persisting bacterial population seems to be unchanged, as well as when predominant strains take over each other. To elucidate the different or similar mechanisms behind these two presumably distinct strategies of phage resistance development, we plan to further study these strains. PFGE is a convenient and cost-effective method for preliminary genetic screening of a pool of strains, but for drawing any conclusions about the mechanisms of adaptation, whole genome sequencing is required. In particular, if we consider the fact that strain’s persistence properties can be determined even by single nucleotide polymorphisms [22].

While phages do have some pharmacological limitations and their production is still hindered by regulatory obstacles, they are undeniably considered an alternative to failing antibiotics and phage production for mass consumption is anticipated [26,27]. However, offering custom phages to patients is diverting this field more towards individualized medicine, which, globally, is still a goal rather than an achievement, especially considering the ever-growing data on the human microbiome [28]. Therefore, the question that the authors of this article want to bring to the public’s attention is whether phage therapy should even go another step ahead by adjusting phage treatment strategies according to each patient’s very specific conditions, such as the composition, diversity and adaptation potential of their bacterial populations. It is clear that mass production of readily available predefined phage preparations will favor accessibility to phages. However, if individualized approach is prioritized, the field might benefit greatly by incorporating a possible management plan for eventual events of phage resistance.

## Figures and Tables

**Table 1:** Four phage preparations used in this study. Listed preparations are manufactured by Eliava Biopreparations. Bacterial species targeted by these readily available preparations are shown in column 2. The titer of each phage component in these preparations is 10^5^ PFU/mL except for Streptococcal phages (10^4^ PFU/mL) and for the Staphylococcal phage in the Staphylococcal Bacteriophage preparation (10^7^ PFU/mL).

Phage Preparation	Active against	Recipients from This Study
Pyo Bacteriophage	*Escherichia coli*, *Proteus mirabilis*, *Proteus vulgaris*, *Pseudomonas aeruginosa*, *Staphylococcus aureus*, *Streptococcus pyogenes*, *Streptococcus mitis.*	Patient #1
Intesti Bacteriophage	*Escherichia coli*, *Enterococcus faecalis*, *Proteus mirabilis*, *Proteus vulgaris*, *Pseudomonas aeruginosa*, *Salmonella enterica* subsp. *Enterica* (serotypes: Choleraesuis, Enteritidis, Oranienburg, Paratyphi, Typhimurium), *Shigella flexneri*, *Shigella sonnei, Staphylococcus aureus.*	Patients #1, #3
SES Bacteriophage	*Escherichia coli* (EPEC), *Staphylococcus aureus*, *Staphylococcus epidermidis*, *Streptococcus pyogenes*, *Streptococcus salivarius*, *Streptococcus sanguinis.*	Patient #3
Staphylococcal Bacteriophage	*Staphylococcus aureus.*	Patient #2

**Table 2:** Phage susceptibility of *Pseudomonas aeruginosa* sputum cultures of patient #1: strain sensitive to phage (S), intermediate sensitivity (I), strain resistant to phage (R). * Custom phage susceptibility of strains isolated before custom phage preparation. ** Bacterial strains isolated in or after 2020 were not included in the study.

Sputum Culture Collection Date	Susceptibility to Pyo phage	Susceptibility to Intesti Phage	Susceptibility To Custom Phage	Also Present
9 January 2017	S	S	R *	N/A
24 January 2017	S	S	R *	*S. mitis*
3 April 2017a	I	I	S *	*S. aureus*, *S. mitis*
3 April 2017b	I	I	R *	*S. aureus*, *S. mitis*
23 September 2017	I	I	R *	N/A
17 September 2018	I	I	R *	*S. mitis*
15 January 2019	R	R	S *	*S. chromogenes*, *S. mitis*
February 2019	Ordered custom phage			
1 October 2019	I	I	R	*S. aureus*
23 September 2020 **	I	I		*S. epidermidis*, *S. oralis*

**Table 3:** Phage susceptibility of *Pseudomonas aeruginosa* sputum (all samples except for the one collected on 19 June 2018) and nose swab (collected on 19 June 2018) cultures of patient #2: strain sensitive to phage (S), strain resistant to phage (R). Two strains of *Pseudomonas aeruginosa* were isolated from morphologically distinct colonies (31 July 2019a—greenish blue colony variants, 31 July 2019b—pale pinkish colony variants) found in the same sputum sample. * Custom phage susceptibility of strains isolated before custom phage preparation. ** Custom phages and bacterial strains from 2020 or later were not included in the study.

Culture Collection Date	Susceptibility to Pyo Phage	Susceptibility to Intesti Phage	Susceptibility to Custom Phage 1	Susceptibility to Custom Phage 2	Susceptibility to Custom Phage 3	Also Present
18 June 2018	R	R	R *	S *	S *	N/A
19 June 2018	R	R	S *	R *	R *	*S. aureus*
July 2018	Ordered Custom phage 1					
2 July 2018	No *P. aeruginosa* present		N/A	N/A	N/A	*S. aureus*, *S. oralis*, *R. pickettii*
19 November 2018	R	R	R	R	R	N/A
11 March 2019	R	R	S	S	S	*S. aureus*
April 2019	Ordered Custom phage 2. Custom phage 2 was adapted to strain isolated on 11 March 2019.					
31 July 2019a	R	R	S	S	S	*S. oralis*
31 July 2019b	R	R	R	R	R	*S. oralis*
September 2019	Ordered Custom phage 3. Custom phage 3 was adapted to strain isolated on 31 July 2019.					
2 September 2019	R	R	R	R	R	N/A
29 January 2020 **	R	R				*S. oralis*
February 2020 **	Ordered Custom phage 4					
8 August 2020 **	R	R				N/A
September 2020 **	Ordered Custom phage 5					
10 February 2021 **	R	R				*S. oralis*
July 2021 **	No *P. aeruginosa* present					

**Table 4:** Urine and vaginal swab cultures of patient #3: strain sensitive to phage (S), strain resistant to phage (R). Phage susceptibility marked with asterisks applies to the strains that were isolated before custom phage preparation.

Culture Collection Date (Source)	*K. pneumoniae* Present:	Susceptibility to Custom Phage 1	Susceptibility to Custom Phage 2	Also Present
8 January2018 (urine)	Yes	R	S *	N/A
26 March 2018 (urine)	Yes	S *	S *	N/A
March 2018	Ordered custom phage 1			
26 June 2018 (urine)	Yes	R	S *	*E. faecalis*
26 June 2018 (vaginal swab)	Yes	R	S *	*E. faecalis*, *E. coli*
6 July 2018 (urine)	No	N/A	N/A	*E. faecalis*
9 July 2018 (urine)	Yes	R	S *	N/A
9 October 2018 (urine)	Yes	R	S *	N/A
9 October 2018 (vaginal swab)	Yes	R	R *	*E. faecalis*, *S. epidermidis*
9 January 2019 (urine)	Yes, 2 strains	R,S	S *, S *	N/A
9 January 2019 (vaginal swab)	Yes, 2 strains	R,S	S *, S *	*E. faecalis*
January 2019	Ordered custom phage 2. Custom phage 2 was adapted to strains isolated on 9 January 2019.			
23 April 2019 (urine)	Yes	R	R	N/A
23 April 2019 (vaginal swab)	Yes	R	S	*E. faecalis*
12 July 2019 (urine)	Yes	R	S	N/A
12 July 2019 (vaginal swab)	Yes	R	R	*E. coli*

**Table 5:** A short summary of three patient cases and their treatment plans discussed in this work.

	Patient #1	Patient #2	Patient #3
Gender	male	female	female
Age	43	64	72
Diagnosis	Cystic fibrosis	Primary ciliary dyskinesia, bronchiectasis	Chronic cystitits, bacterial vaginitis
Main causative agent	*P. aeruginosa*	*P. aeruginosa*	*K. pneumoniae*
Custom phage titer	9 × 10^6^–1 × 10^7^ PFU/mL	4 × 10^6^–6 × 10^6^ PFU/mL (phages 1–5)	8 × 10^6^ PFU/mL (phage 1), 7 × 10^8^ (phage 2)
Route of custom phage administration	Oral, inhalation via nebulizer	Oral	Oral, vaginal suppositories
Other phage preparations included in the treatment plan	Pyo, Intesti	Staophylococcal phage	Intesti, SES
Antibiotics included in the treatment plan	None *	None	vaginal suppositories containing metronidazole, miconazole, extract of Centella asiata, polymixin B and neomycin
Total duration of phage therapy	January 2017–February 2021	September 2018–present	June 2018–June 2019

* The patient took antibiotics without EPTC doctors’ involvement or supervision in 2019.

## Data Availability

The data presented in this study are available in the article and in Supplementary Material.

## Supplementary Materials

The following are available online at https://www.mdpi.com/article/10.3390/v13101901/s1, Figure S1: Fragments of two PFGE gel pictures of *Spe*I digested total DNA of *P. aeruginosa* strains of patient #1, Figure S2: Fragments of two PFGE gel pictures of SpeI digested total DNA of *P. aeruginosa* strains of patient #2, Figure S3: PFGE gel picture of XbaI digested total DNA of K. pneumoniae strains of patient #3; Table S1: Bacterial load in bacteriological samples of patient #1 is given in colony forming units (CFU) per mL, Table S2: Antibiotic susceptibility information for *P. aeruginosa* strains of patient #1, Table S3: Bacterial load in bacteriological samples of patient #3 is given in colony forming units (CFU) per mL, Table S4: Bacterial load in bacteriological samples of patient #2 is given in colony forming units (CFU) per mL. Table S5: Antibiotic susceptibility information for *K. pneumoniae* strains of patient #3.
