# Supplementary material for: Phage Therapy Experience at the Eliava Phage Therapy Center: Three Cases of Bacterial Persistence
Source: Viruses. 2021 Sep 23;13(10):1901. doi: 10.3390/v13101901 (PMC8540005; doi:10.3390/v13101901)
Supplement: Supplementary file 1 [file viruses-13-01901-s001.zip › viruses-1360491-supplementary.pdf]

Phage therapy experience at the Eliava Phage Therapy Center: three cases of bacterial persistence: Supplementary material

Determination of bacterial load (CFU/ml) in each specimen is a part of routine bacteriological analysis done at Eliava Analytical-Diagnostic Center. This information is then provided to the clinicians.

Table S1. Bacterial load in bacteriological samples of patient #1 is given in colony forming units (CFU) per mL.

Culture collection date	*P. aeruginosa*	Other bacteria
09.01.2017	$5x10^5$ CFU/ml	N/A
24.01.2017	$5x10^4$ CFU/ml	*S. mitis*: $1x10^8$ CFU/ml
03.04.2017	$1x10^5$ CFU/ml	*S. mitis*: $5x10^6$ CFU/ml, *S. aureus*: $1x10^6$ CFU/ml
23.09.2017	$1x10^6$ CFU /ml	S. aureus: $5x10^6$ CFU/ml
17.09.2018	$1x10^6$ CFU/ml	*S. mitis*: $1x10^6$ CFU/ml
15.01.2019	$1x10^6$ CFU/ml	*S. mitis*: $1x10^5$ CFU/ml, *S. chromogenes*: $1x10^6$ CFU/ml
01.10.2019	$1x10^4$ CFUml	*S. aureus*: $5x10^5$ CFU/ml

Table S2. Antibiotic susceptibility information for *P. aeruginosa* strains of patient #1. Only those antibiotics are shown, to which changes in susceptibility have been observed.

Strain collected on	Cefepime	Meropenem	Ciprofloxacin	Levofloxacin	Amikacin	Tobramycin	Netilmicin
09.01.2017	S	S	R	R	I	R	R
24.01.2017	R	S	R	R	R	R	R
03.04.2017a	S	S	S	S	S	S	S
03.04.2017b	S	S	R	R	R	R	R
23.09.2017	S	I	R	R	S	S	S
17.09.2018	S	S	R	R	S	S	S
15.01.2019	S	S	S	S	S	S	S
01.10.2019	S	S	R	R	S	S	S

Table S3. Bacterial load in bacteriological samples of patient #3 is given in colony forming units (CFU) per mL.

Culture collection date (source)	*K. pneumoniae*	Other bacteria
08.01.2018 (urine)	Information unavailable	
26.03.2018 (urine)	$1x10^8$ CFU/ml	N/A
26.06.2018 (urine)	$1x10^6$ CFU/ml	*E. faecalis*: >$1x10^3$ CFU/ml
26.06.2018 (vaginal swab)	$3x10^3$ CFU/ml	*E. faecalis*: $1x10^5$ CFU/ml, *E. coli*: $5x10^4$ CFU/ml
06.07.2018 (urine)	Not present	*E. faecalis*: >$1x10^3$ CFU/ml
09.07.2018 (urine)	$1x10^6$ CFU/ml	N/A
09.10.2018 (urine)	$5x10^6$ CFU/ml	N/A
09.10.2018 (vaginal swab)	$1x10^8$ CFU/ml	*S. epidermidis*: $5x10^6$ CFU/ml, *E. faecalis*: $1x10^8$ CFU/ml
09.01.2019 (urine)	$5x10^6$ CFU/ml	N/A

09.01.2019 (vaginal swab)	$1x10^5$ CFU/ml	*E. faecalis*: $1x10^5$ CFU/ml
23.04.2019 (urine)	$1x10^6$ CFU/ml	N/A
23.04.2019 (vaginal swab)	$5x10^4$ CFU/ml	*E. faecalis*: $5x10^4$ CFU/ml
12.07.2019 (urine)	$1x10^8$ CFU/ml	N/A
12.07.2019 (vaginal swab)	$1x10^8$ CFU/ml	*E. coli*: $1x10^8$ CFU/ml

Disc diffusion methodology was employed for antibiotic susceptibility testing. Selection of antibiotic discs as well as interpretation of the results was done according to latest EUCAST recommendations. R – resistance, I – intermediate sensitivity, S – sensitivity.

Table S4. Bacterial load in bacteriological samples of patient #2 is given in colony forming units (CFU) per mL.

Culture collection date	*P. aeruginosa*	Other bacteria
18.06.2018	$1x10^6$ CFU/ml	N/A
19.06.2018	$1x10^5$ CFU/ml	*S. aureus*: >$1x10^3$ CFU/ml
02.07.2018	No *P.aeruginosa* present	*Ralstonia pickettii*: $1x10^6$ CFU/ml, *Staphylococcus aureus*: $3x10^3$ CFU/ml, *Streptococcus oralis*: $3x10^3$ CFU/ml
19.11.2018	$5x10^6$ CFU/ml	N/A
11.03.2019	$5x10^6$ CFU/ml	*S. aureus*: $3x10^3$ CFU/ml
31.07.2019	$1x10^6$ CFU/ml	*S. oralis*: $5x10^4$ CFU/ml
02.09.2019	$1x10^5$ CFU/ml	N/A

Table S5. Antibiotic susceptibility information for *K. pneumoniae* strains of patient #3. Only those antibiotics are shown, to which changes in susceptibility have been observed. (u) – urine isolates, (v) – vaginal isolates.

Strain collected on (source)	Ertapenem	Meropenem	Aztreonam	Tobramycin	Chloramphenicol	Trimethoprim	Trimethoprim /sulfamethoxa sole
08.01.2018 (u)	R	I	R	S	S	S	S
26.03.2018 (u)	S	S	R	I	R	R	R
26.06.2018 (u)	S	S	R	I	R	R	R
26.06.2018 (v)	S	S	R	I	R	R	R
09.07.2018 (u)	S	S	R	R	R	R	R
09.10.2018 (u)	R	I	R	I	S	R	R
09.10.2018 (v)	S	S	R	R	S	R	R
09.01.2019 (u)	R	I	R	S	S	I	S
09.01.2019 (u)	R	I	R	S	S	S	S
09.01.2019 (v)	S	I	I	I	S	R	R
09.01.2019 (v)	R	I	R	R	S	R	R
23.04.2019 (u)	R	I	R	I	R	R	R
23.04.2019 (v)	R	I	I	I	S	R	R
12.07.2019 (u)	R	S	R	S	R	S	S
12.07.2019 (v)	R	I	R	S	R	I	S

No changes in antibiotic susceptibility were observed in any of the *P. aeruginosa* strains of patient #2.

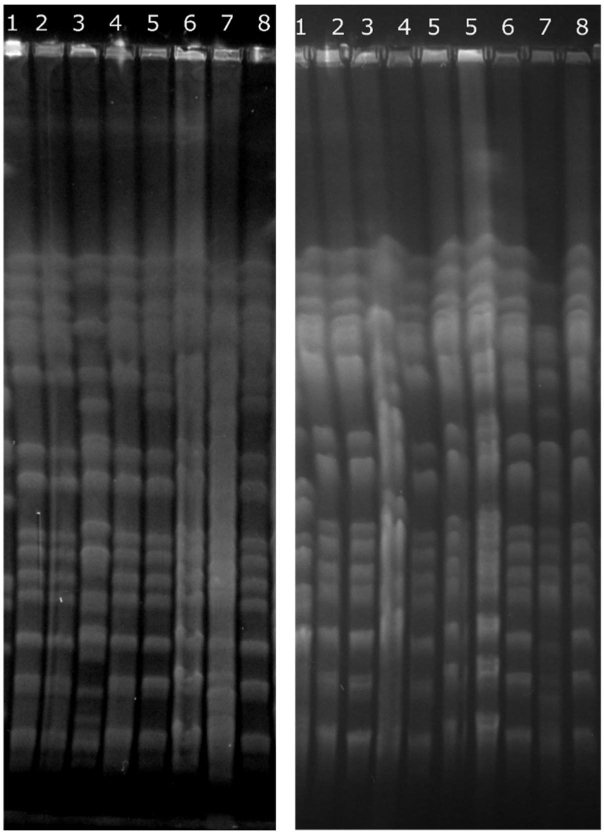

Figure S1. Fragments of two PFGE gel pictures of *Spe*I digested total DNA of *P. aeruginosa* strains of patient #1. 1-8: *P. a*. sputum isolates from 09.01.2017 (1), 24.01.2017 (2), 03.04.2017 (3), 03.04.2017 (4), 23.09.2017 (5), 17.09.2018 (6), 15.01.2019 (7) and 01.10.2019 (8).

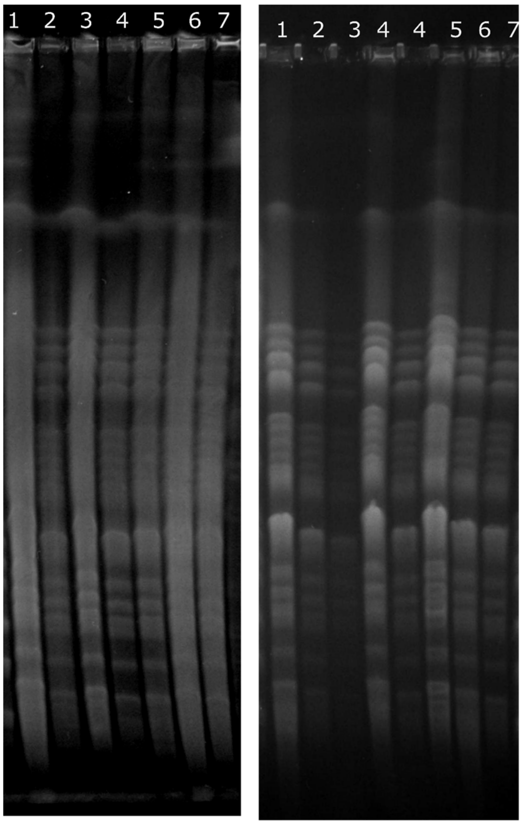

Figure S2. Fragments of two PFGE gel pictures of *Spe*I digested total DNA of *P. aeruginosa* strains of patient #2. 1, 3-7: *P. a.* sputum isolates from 18.06.2018 (1), 19.11.2018 (3), 11.03.2019 (4), 31.07.2019a (5), 31.07.2019b (6) and 02.09.2019 (7). 2: *P. a.* nose swab isolate from 19.06.2018.

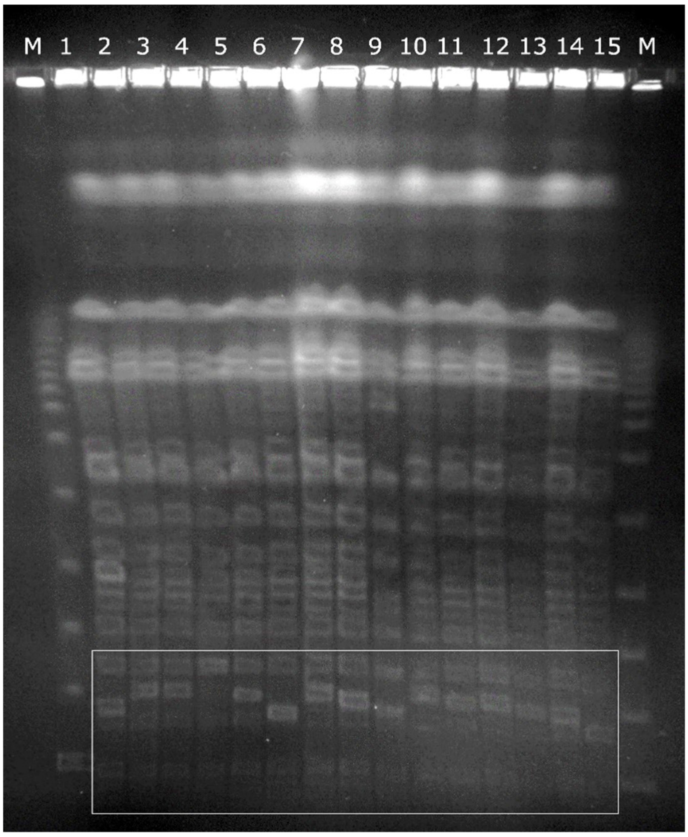

Figure S3. PFGE gel picture of *Xba*I digested total DNA of *K. pneumoniae* strains of patient #3. M: Lambda PFG ladder (NEB); 1-9: *K. p*. urine isolates from 08.01.2018 (1), 26.03.2018 (2), 26.06.2018 (3), 09.07.2018 (4), 09.10.2018 (5), 09.01.2019(6), 09.01.2019 (7), 23.04.2019 (8) and 12.07.2019 (9); 10-15: *K. p*. vaginal isolates from 26.06.2018 (10), 09.10.2018 (11), 09.01.2019 (12), 09.01.2019 (13), 23.04.2019 (14) and 12.07.2019 (15). Main differences stemmed from low molecular weight fragments (boxed).
